# CD70/CD27 signaling promotes the pathogenesis of multiple myeloma and represents a promising therapeutic target

**DOI:** 10.1038/s41375-026-02899-1

**Published:** 2026-03-23

**Authors:** Stefan Forster, Chantal Reinhardt, Maxime Boy, Adrian Wegmüller, Alessio Hinrichsen, Christian M. Schürch, Frido K. Brühl, Falko Fend, Alexandar Tzankov, Marie-Noëlle Kronig, Michaela Römmele, Angéline Glück, Benjamin Lüscher, Myriam Legros, Ulrike Bacher, Katja Seipel, Thomas Pabst, Ramin Radpour, Carsten Riether, Adrian F. Ochsenbein

**Affiliations:** 1 Tumor Immunology, Department for BioMedical Research (DBMR), University of Bern, Bern, Switzerland; 2 Department of Medical Oncology, Inselspital, Bern University Hospital, University of Bern, Bern, Switzerland; 3 Institute of Pathology, Technical University Munich, Munich, Germany; 4 Department of Pathology and Neuropathology, University Hospital and Comprehensive Cancer Center Tübingen, Tübingen, Germany; 5 Cluster of Excellence iFIT (EXC 2180) “Image-Guided and Functionally Instructed Tumor Therapies”, University of Tübingen, Tübingen, Germany; 6 Department of Laboratory Medicine and Pathology, Ohiohealth, Columbus, OH USA; 7 Institute of Medical Genetics and Pathology, University Hospital Basel, Basel, Switzerland; 8 Clinical Genomics Lab, Inselspital, Bern University Hospital, University of Bern, Bern, Switzerland; 9 Department of Hematology and Central Hematology Laboratory, Inselspital, Bern University Hospital, University of Bern, Bern, Switzerland

**Keywords:** Myeloma, Immunotherapy

## Abstract

The increasing therapeutic options for multiple myeloma (MM) have significantly improved long-term survival for many patients. However, disease progression remains inevitable due to the emergence of drug-resistant myeloma cell populations, often culminating in extramedullary disease manifestations. In this study, we identified CD70-expressing myeloma cells as critical drivers of disease progression and propagation. CD70 expression in MM cells is an independent negative prognostic factor for overall survival. Mechanistically, CD70/CD27 signaling activates the MAPK/ERK and Wnt signaling pathways in MM cell lines and primary patient-derived MM samples, promoting increased cell cycling and proliferation. This proliferative advantage results in elevated CD70 expression in advanced MM stages, particularly in extramedullary myeloma. Functional inhibition of CD70/CD27 signaling, achieved either by generating CD70-knockout primary MM cells or with blocking monoclonal antibodies, completely abrogated tumor growth in xenotransplantation models. Furthermore, the ADCC-enhanced anti-CD70 antibody, cusatuzumab, demonstrated high efficacy in treating myeloma in xenotransplantation models. Collectively, these findings underscore the critical role of CD70/CD27 signaling in activating MAPK/ERK and Wnt pathways essential for MM progression. Targeting CD70 with blocking or ADCC-enhanced antibodies represents a promising therapeutic strategy, particularly for advanced MM stages characterized by high CD70 expression.

## Introduction

Multiple myeloma (MM) is characterized by the uncontrolled proliferation of malignant plasma cells within the bone marrow (BM). Clinical manifestations include pathological bone fractures and kidney impairment caused by the rapid division of plasma cells and the release of abnormal immunoglobulins [1–4]. Over the past decade, substantial progress in MM treatment has significantly improved progression-free and overall survival (OS) rates [5]. In addition to proteasome inhibitors and immunomodulatory drugs, novel and innovative immunotherapies targeting plasma cell surface molecules have become a cornerstone in treating MM, particularly in patients experiencing relapse or refractory disease stages [6–10].

Despite these advancements, MM remains incurable, with nearly all patients eventually succumbing to disease relapse [11]. A major challenge in achieving complete eradication of myeloma cells is the high intratumoral heterogeneity, characterized by the coexistence of multiple clonal subpopulations [12].

CD70 (TNFSF7) and CD27 (TNFRSF7) belong to the tumor necrosis factor (TNF) receptor family, where CD70 is the only known ligand for its receptor CD27 [13]. Physiologically, the CD70/CD27 signaling pathway regulates adaptive immune responses by promoting lymphocyte activation and memory formation [14, 15]. Furthermore, this interaction plays a pivotal role in B lymphocyte differentiation into plasma cells [16]. Upon CD70 ligation, CD27 is cleaved from the cell surface, producing a soluble form (sCD27) detectable in body fluids, such as serum [17, 18].

CD70 expression has been linked to disease progression and serves as a negative prognostic marker in various malignancies, including renal cancer, glioblastoma, and osteosarcoma [19–21]. Co-expression of CD70 and CD27 has been reported in several hematologic malignancies, such as acute myeloid leukemia (AML) and Waldenström macroglobulinemia [22, 23]. In AML, CD70/CD27 signaling drives self-renewal and expansion of leukemic stem cells [22]. In MM, CD70 expression has been observed in 40% of patients [24]. However, flow cytometric analysis of BM aspirates has revealed that only in 3% of MM patients more than 20% of BM-infiltrating plasma cells express CD70 [25]. CD27 expression on malignant plasma cells has been reported in approximately 44% of MM patients at diagnosis, with levels declining as the disease progresses [26–28]. In contrast, elevated CD27 expression has been noted in primary plasma cell leukemia, where CD70-induced CD27 activation prevents both spontaneous and dexamethasone-induced apoptosis [29].

In this study, we demonstrate that CD70 is expressed on a subset of MM cells during early disease stages, which exhibit higher proliferative and disease-initiating capacities. CD70 expression increases as the disease progresses, reaching its maximum in extramedullary myeloma. CD70/CD27 signaling promotes disease initiation and clonal plasma cell expansion through the induction of MAPK/ERK and Wnt signaling. Furthermore, we show that targeting CD70 with monoclonal antibodies (mAbs) effectively eliminates CD70-expressing myeloma cells both in vitro and in vivo.

## Materials and Methods

### Mice

Male and female NOD.Cg-PrkdcscidIl2rgtm1Wjl/SzJ (NSG) mice were purchased at an age between 6 – 8 weeks from Charles River Laboratories. Mice were housed under specific pathogen-free conditions in ventilated cages with food and water supply ad libitum. All mice were regularly monitored for pathogens. No randomisation procedures were used in the allocation of animals to experimental groups. Experiments were conducted with age- and sex matched animals in a non-blinded fashion, approved by the local experimental animal committee of the Canton of Bern. All experiments were performed according to the Swiss laws for animal protection (BE12/21).

### MM patient material and MM patient-derived xenografts

MM primary tissue specimens were obtained from patients with informed consent at the University Hospital Bern, Switzerland. Patient-derived myeloma tissues were cut into small pieces of about 5mm in side length. Afterwards, pieces were subcutaneously implanted into the flanks of 6-8-weekold NSG mice. As soon as the xenotransplants reached a maximum size of 1000mm^3^, tumors were resected, and single cells were isolated through mechanical tissue disruption. For serial injections, CD70+ and CD70- cells were purified using a MoFlo ASTRIOS BSL-2 cell sorter (Beckman Coulter). Sorted cells were then injected in Hank’s Balanced Salt Solution (HBSS) into the flanks or the tibial bones of 6-8week-old NSG mice. For treatments, mice were intraperitoneally injected with 10 mg/kg of 41D12-D, cusatuzumab or the respective control antibodies every third day starting from the day of myeloma cell injection or when engrafted tumors reached an average volume of 150mm^3^. Subcutaneous tumor volumes were measured in 3 dimensions with a caliper. For calculation of tumor volumes, the formula volume (v) = π/6 × length × width × width was used.

A detailed description of materials and methods is in the Supplemental Data.

## Results

### High *CD70* expression is a predictor of poor clinical outcomes in MM

**Fig. 1:**
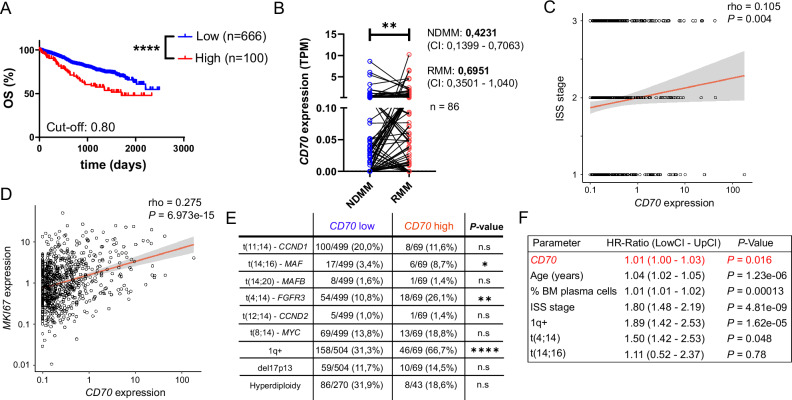
High *CD70* gene expression is linked to poor myeloma outcomes. **A** Kaplan-Meier survival curve of *CD70* high (*n* = 100) versus *CD70* low (*n* = 666) MM patients (gene expression data were extracted from the CoMMpass database; identifier IA15). **B** Longitudinal gene expression analysis of *CD70* in matched newly diagnosed MM (NDMM) and relapsed MM (RMM) samples (*n* = 86; gene expression data were extracted from the CoMMpass database; identifier IA22). **C**, **D** Correlation analyses between international staging system (ISS) stages (**C**), or *MKI67* gene expression levels (**D**) and *CD70* expression (data were extracted from the CoMMpass database; identifier IA15). **E** Percentages of selected cytogenetic abnormalities in *CD70* high versus *CD70* low myeloma patients enrolled in the CoMMpass study (identifier IA15). **F** Multivariate analysis for *CD70* adjusted for ISS risk groups, age, percentage (%) of BM infiltrating plasma cells and high-risk cytogenetic alterations. Statistics: log-rank test (**A**), Wilcoxon matched-pairs signed rank test (**B**), Spearman’s rho test (**C**, **D**), Chi-squared test (**E**), multiple cox regression (**F**); *, *P* < 0.05; **, *P* < 0.01; ****, *P* < 0.0001. Data are shown as mean with standard deviation (SD).

To address the prognostic impact of CD70 in MM, we analyzed the CoMMpass gene database (version IA15), which includes RNA sequencing (RNA-seq) data of purified CD38⁺ CD138⁺ BM plasma cells from 766 MM patients at diagnosis. We first stratified patients by median *CD70* expression and correlated expression levels with patients’ overall survival (OS). In line with previously published data from Kasap et al., a significantly worse OS in the *CD70* high compared to the *CD70* low group was observed [30] (Supplemental Fig. 1A). In addition, we applied maximally selected rank statistics, which revealed the greatest survival differences at a cut-off point of 0.8 TPM, separating 13.05% of patients into *CD70* high and 86.95% of patients into *CD70* low groups. Of note, this survival difference was very robust and hold true over a broad range of cut-points between *CD70* high and *CD70* low groups, including the median (Fig. 1A and Supplemental Fig. 1B). To investigate *CD70* expression during MM progression, *CD70* expression levels were compared between 86 matched newly diagnosed and relapsed disease samples from the MMRF CoMMpass database (version IA22). *CD70* expression was significantly increased in relapsed MM, suggesting upregulation of CD70 during disease progression (Fig. 1B). Based on data extracted from the CoMMpass database, *CD70* expression showed a weak but significant correlation with known adverse prognostic markers, including International Staging System (ISS) risk group, lactate dehydrogenase (LDH) and β2-microglobulin serum levels as well as *MKI67* gene expression levels, suggesting higher disease activity in *CD70* high patients (Fig. 1C, D and Supplemental Fig. 1C, D). We also examined the association between *CD70* expression and high-risk molecular and cytogenetic aberrations in MM. *CD70* was found to be more frequently expressed in patients with high-risk genomic translocations, including t(4;14) and t(14;16), as well as gain or amplification of the chromosomal region 1q21 (+1q) (Fig. 1E). To determine whether *CD70* expression serves as an independent prognostic marker, we additionally performed multivariate analyses, adjusting for patient’s age, ISS risk group, percentage of BM-infiltrating plasma cells and adverse cytogenetic alterations. The results confirmed *CD70* expression as a significant independent prognostic marker in MM patients (Fig. 1F).

In summary, our findings demonstrate that *CD70* gene expression is associated with high-risk cytogenetic alterations, poor prognosis, and advanced disease stages in MM.

### CD70 is frequently expressed in relapsed and therapy-refractory extramedullary disease stages

**Fig. 2:**
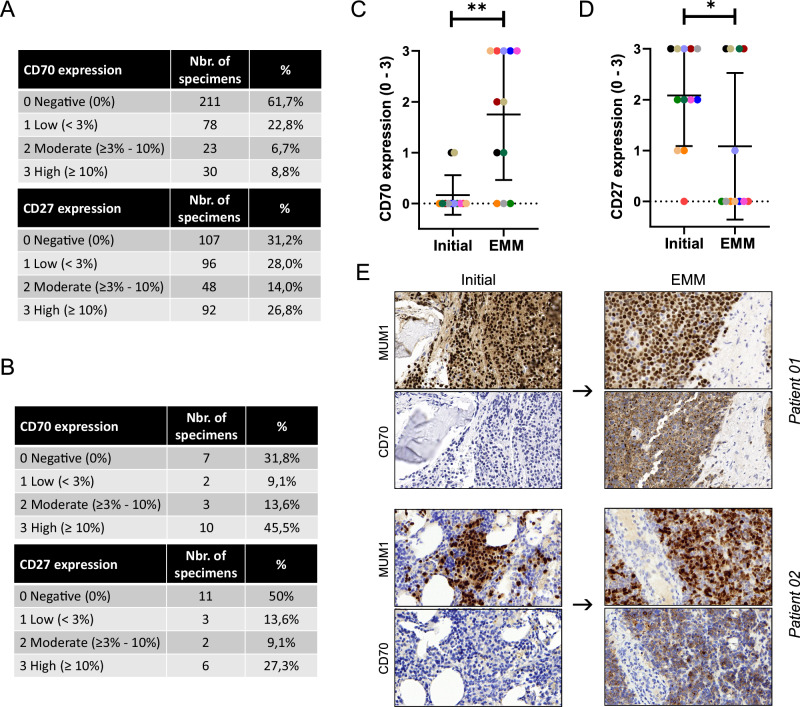
CD70 and CD27 expression in early and advanced myeloma stages. **A** TMA-based evaluation of CD70 and CD27 protein expression profiles in MM patients at early disease stages. CD70 (*n* = 342 biopsies) and CD27 (*n* = 343 biopsies) protein expression levels were scored based on the percentages of CD70 or CD27 positive infiltrating plasma cells. **B** CD70 and CD27 expression levels were scored on whole tissue slides (WTS) of extramedullary myeloma (EMM) and plasma cell leukemia (PCL) (total *n* = 22). **C** CD70 expression between matched biopsies at initial diagnosis and relapse with manifestation of extramedullary disease (EMM) (*n* = 12). **D** CD27 expression between paired samples from initial manifestation and EMM (*n* = 12). **E** Representative images of matched MM specimens from two individual patients (Patient 01 and Patient 02) showing CD70 expression on MUM1 positive myeloma cells at the initial diagnosis and relapse with EMM. Statistics: Wilcoxon matched-pairs signed rank test (C, D); *, *P* < 0.05; **, *P* < 0.01. Data are shown as mean with SD.

We next examined CD70 and CD27 protein expression levels in a tissue microarray (TMA) dataset containing samples from early-stage disease. The expression levels were scored based on the frequency of CD70- or CD27-positive myeloma cells in 342 and 343 MM specimens, respectively (Supplemental Fig. 2A). Consistent with a previous smaller study, we observed that CD70 was expressed in 38.3% of all MM specimens [24]. Notably, 8.8% of the biopsies showed ≥10% CD70-positive plasma cells. In 40.8% of the specimens, ≥3% of plasma cells expressed CD27, and in 26.8%, CD27 expression was detected in ≥10% of malignant plasma cells (Fig. 2A). Additionally, CD70 and CD27 protein levels were analyzed in 22 extramedullary myeloma (EMM) samples. CD70 expression was substantially higher in EMM, suggesting increased CD70 expression in advanced disease stages (Fig. 2B). To further validate our previous findings from the MMRF database, we longitudinally analyzed 12 relapsed EMM samples and their matched BM biopsies from the time of initial diagnosis. This analysis revealed a significant increase in CD70 expression in relapsed EMM stages, whereas CD27 expression was significantly reduced in EMM compared to the initial MM diagnosis (Fig. 2C–E).

Since the majority of MM cell lines are derived from EMM stages, we also examined *CD70* and *CD27* gene expression in a panel of 29 MM cell lines using publicly available data from the Cancer Cell Line Encyclopedia (CCLE) initiative [31]. Additionally, we evaluated CD70 and CD27 surface expression by flow cytometry on 7 MM cell lines (U266, RPMI8226, SK-MM1, LP1, L363, KMS12-BM, and MOLP2). High CD70 gene and protein expression were detected in most MM cell lines (Supplemental Fig. 2B, C), while CD27 gene and protein expression were either low or absent (Supplemental Fig. 2B, D). CD70 and CD27 co-expression was observed in the MM cell lines KMS12-BM and MOLP2.

In summary, our data suggest that CD70 expression increases during the progression of MM, with the highest levels observed in EMM.

### Hypoxia induces CD70 expression in myeloma cells

**Fig. 3:**
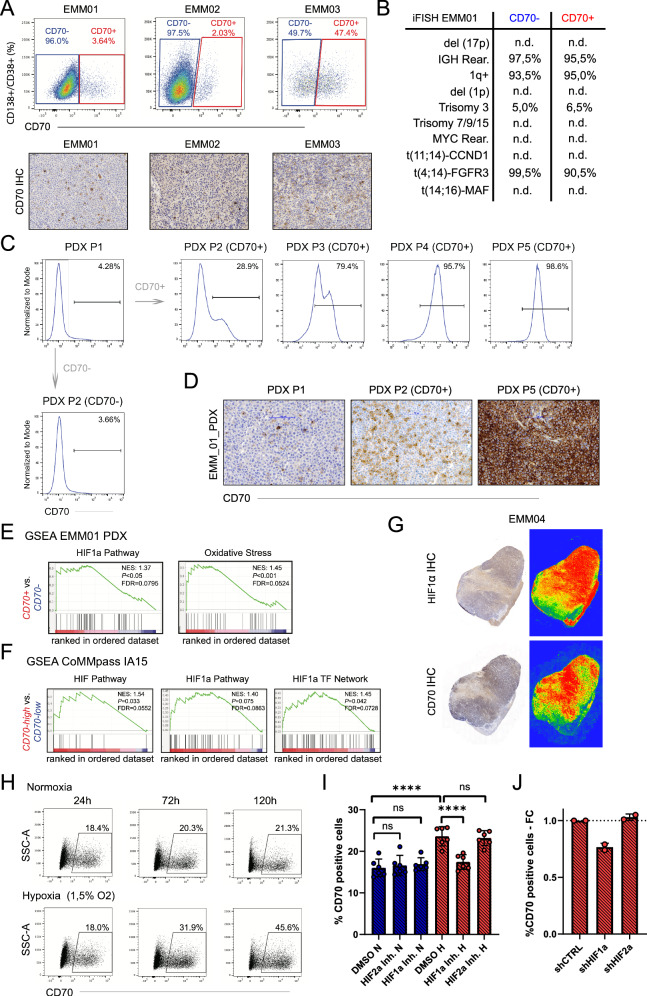
Hypoxia causes CD70 upregulation in MM. **A** Detection of CD70+ and CD70- myeloma cell subsets in patient-derived xenografts (PDXs) from three patients with advanced MM stages (EMM01, EMM02 and EMM03) confirmed by flow cytometry (upper panel) and immunohistochemistry (IHC) staining (lower panel). **B** Interphase fluorescence in situ hybridization (iFISH) analysis for distinct cytogenetic alterations in EMM01-derived CD70+ and CD70- myeloma cell fractions (n.d.; not detected). **C**, **D** Comparison of CD70 expression analyzed by flow cytometry (**C**) and IHC (**D**) between EMM01 parental (passage 1; P1) and CD70 + EMM01 xenografts after serial injections of CD70+ myeloma cells in NSG mice; injection of CD70- fractions resulted in detection of a CD70+ subset in successfully grown xenografts. **E** Gene set enrichment analysis (GSEA) of HIF1a pathway and oxidative stress - related genes in CD70+ vs. CD70- fractions isolated from our established EMM01 PDX. **F** GSEA of genes involved in HIF/HIF1a mediated signaling pathways in *CD70* high versus *CD70* low MM patients enrolled in the CoMMpass study (identifier IA15). **G** Spatial expression profiles of CD70 and HIF1a evaluated in one MM specimen with infiltration of CD70+ myeloma cells (EMM04). Areas with high CD70 and HIF1a expression are highlighted using SlideViewer heatmap. **H** CD70 expression on SK-MM1 cells cultured in normoxic and hypoxic conditions (1.5% O2) over 24 h, 72 h and 120 h; one out of tree independent experiments is shown. **I** CD70 expression of SK-MM1 cells treated with HIF1a or HIF2a inhibitors or DMSO control, cultivated under normoxic (N, 21% O2) or hypoxic (H, 1.5% O2) conditions over 72 h. Data from *n* = 6 independent experiments. **J** Fold change (FC) increase of CD70 expressing myeloma cells assessed by flow cytometry in *HIF1A* or *EPAS1*/*HIF2A* knock-down versus control shRNA SK-MM1 cells, cultivated under hypoxic (1.5% O2) conditions over 72 h. Statistics: one-way ANOVA with Tukey’s multiple comparisons test (I); ****, *P* < 0.0001. Data are shown as mean with SD.

We established patient-derived xenografts (PDX) from patients with EMM. These samples co-expressed CD70 and CD27 (Fig. 3A and Supplemental Fig. 3A). To investigate whether CD70+ and CD70- cells represent distinct molecular subclones, we purified both populations by fluorescence-activated cell sorting (FACS) and performed interphase fluorescence in situ hybridization (iFISH) analysis. Identical cytogenetic alterations were observed in both CD70+ and CD70- fractions (Fig. 3B and Supplemental Fig. 3B). Furthermore, next-generation sequencing (NGS) analysis revealed no differences in oncogenic driver mutations between the CD70+ and CD70- fractions from EMM01. Both populations harbored a *BRAF* c.1799 T > A mutation (Supplemental Fig. 3C). These findings indicate that CD70 upregulation reflects subclonal or phenotypic variation rather than distinct genetic clones.

To further study the regulation of CD70 expression, we injected FACS-purified CD70- and CD70+ MM cells from EMM01 subcutaneously into NSG mice. Injection of CD70- cells resulted in the re-emergence of approximately 4% CD70+ cells, resembling the parental population. In contrast, serial re-injections of sorted CD70+ cells in vivo led to an increase in CD70+ cells from 4% to nearly 100% (Fig. 3C, D). This suggests that CD70 expression is dynamically regulated in vivo and confers a growth advantage to CD70 + MM cells, enabling them to outcompete other populations over time.

Previous studies have reported that hypoxia-inducible factors (HIFs) can induce CD70 expression [32, 33]. Additionally, increased HIF gene expression levels have been observed in EMM [34]. To explore whether hypoxic environmental conditions influence CD70 expression in MM cells, we performed RNA sequencing and conducted gene set enrichment analysis (GSEA) on HIF-related pathways in CD70+ versus CD70- populations derived from the EMM01 PDX sample. CD70+ fractions showed significant enrichment of genes involved in the HIF1α pathway and oxidative stress, suggesting enhanced hypoxia-related signaling (Fig. 3E). Similarly, analysis of *CD70* high versus *CD70* low MM patients from the CoMMpass study revealed significant enrichment of genes in the HIF1α pathway and transcriptional networks, with a strong trend toward HIF1α pathway activity (Fig. 3F). To assess whether CD70 expression is elevated in hypoxic regions of MM samples, we stained an EMM specimen (EMM04) for HIF1α and CD70, enabling spatial comparison of protein expression. The highest expression levels of both CD70 and HIF1α were observed in the tumor center, whereas lower expression was found at the tumor periphery (Fig. 3G). To further investigate the direct effects of hypoxia on CD70 expression, we cultured the MM cell line SK-MM1, characterized by the lowest baseline CD70 surface expression among our screened MM cell lines (Supplemental Fig. 2C), under hypoxic (1.5% O_2_) or normoxic (21% O_2_) conditions for 24, 72 and 120 h. Both CD70 mRNA and protein levels increased under hypoxic conditions (Fig. 3H and Supplemental Fig. 3D). Moreover, hypoxia-induced upregulation of CD70 expression was confirmed in an additional MM cell line RPMI 8226 (Supplemental Fig. 3E). To determine whether HIFs directly induce CD70 expression, we treated SK-MM1 cells with HIF1α or HIF2α inhibitors under normoxic and hypoxic conditions. Inhibition of HIF1α significantly reduced CD70 upregulation under hypoxia, whereas HIF2α inhibition had no effect (Fig. 3I). Similarly, confirmed knockdown of *HIF1A* (shHIF1A) but not *EPAS1/HIF2A* (shHIF2A) using shRNA lentiviral particles, reduced the hypoxia-induced upregulation of CD70 in SK-MM1 cells (Fig. 3J and Supplemental Fig. 3F, G).

In summary, our findings demonstrate that CD70 expression is upregulated in response to environmental cues such as hypoxia, with HIF1α playing a critical role in this process.

### CD70/CD27 signaling promotes the expansion of myeloma cells

To investigate the functional role of CD70/CD27 signaling in MM development, we treated EMM01 CD70+ MM xenotransplanted mice with the anti-CD70 mAb 41D12-D. This antibody blocks the CD70/CD27 interaction but lacks effector functions due to a modification in the CH2 region [35, 36]. Blocking CD70 with 41D12-D led to a significant upregulation of CD27 surface expression on EMM01 CD70+ myeloma cells and a trend towards reduced soluble CD27 (sCD27) serum levels, indicating that the CD70/CD27 interaction and subsequent CD27 shedding were inhibited (Supplemental Fig. 4A, B). Similarly, blocking the CD70/CD27 interaction in vitro with 41D12-D increased CD27 expression in KMS12-BM and MOLP2 MM cells (Supplemental Fig. 4C, D). These findings suggest active CD70/CD27 signaling in CD70-expressing MM, where the interaction contributes to CD27 shedding and reduced surface CD27 expression.

**Fig. 4:**
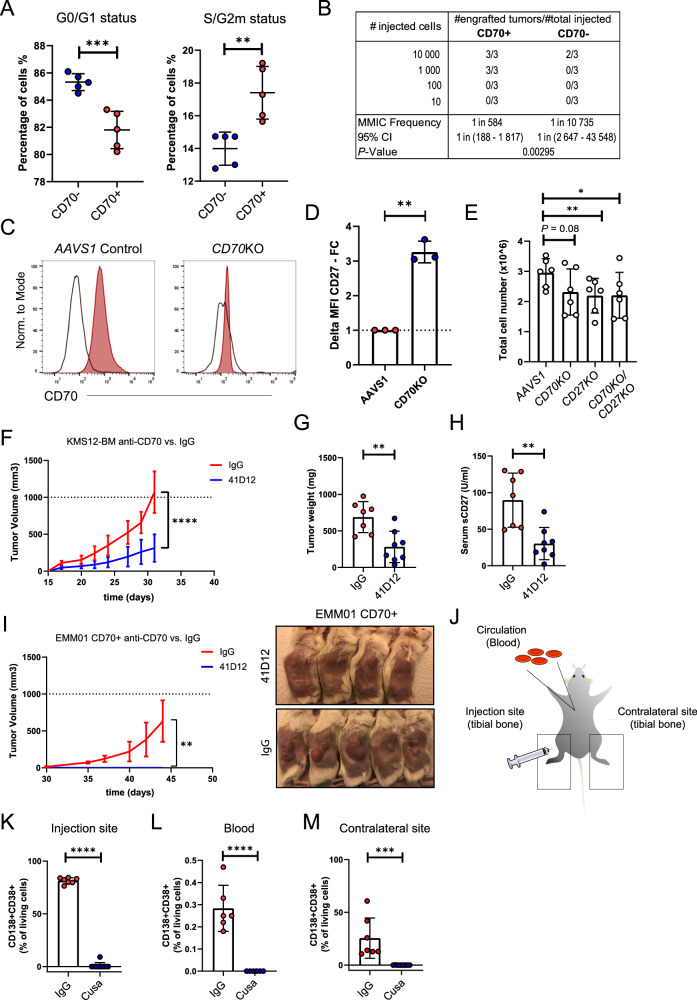
CD70/CD27 signaling activation in MM. **A** Cell cycle states were analyzed in CD70- versus CD70+ myeloma cell fractions using flow cytometry (DAPI) (*n* = 5 / group). **B** In vivo limiting dilution assays were performed by injecting decreasing numbers of EMM01 derived CD70+ or CD70- myeloma cells (10,000–1000–100–10) into the flanks of NSG mice (MMIC; multiple myeloma initiating cells). **C** *CD70* CRISPR-Cas9 knock-out in KMS12-BM MM cell line [70, 71] confirmed by flow cytometry; black: isotype staining; red: CD70 staining antibody. **D** Delta median fluorescence intensity (MFI) of CD27 expression in KMS12-BM AAVS1 or CD70KO cell lines; pooled data from *n* = 3 independent experiments. **E** Total numbers of KMS12-BM AAVS1, CD70KO, CD27KO and CD70KO/CD27KO cells were determined after a 72 h incubation period; pooled data from *n* = 6 independent experiments. **F** Impact of 41D12-D (41D12) or IgG control treatment on MM progression monitored by assessment of tumor volumes of KMS12-BM myeloma bearing mice; treatment was started at the day of injection (d0); data pooled from two independent experiments; *n* = 7 mice / group IgG; *n* = 8 mice / group 41D12. **G**, **H** Impact of 41D12-D (41D12) or IgG control treatments on tumor development (tumor weight measured in mg) (**G**) and soluble CD27 (sCD27) levels (**H**) in KMS12-BM myeloma bearing mice; treatment was started at the day of injection (d0); sCD27 serum levels were measured at day 28 post injection; data pooled from two independent experiments; *n* = 7 mice / group IgG; *n* = 8 mice / group 41D12. **I** 41D12 or IgG control treatments in EMM01 CD70+ injected mice; treatment was started at the day of injection (d0); experiment was performed twice; results from one independent experiment are shown; *n* = 5 (41D12) and *n* = 4 (IgG) mice per group; respective images of mice are shown. **J** EMM01 CD70+ cells were intratibially injected into NSG mice followed by analysis of the primary injection site and sites of MM dissemination (blood and the contralateral tibial bone). **K–M** Cusatuzumab (Cusa) or IgG control treatments of NSG mice that were intratibially injected with EMM01 CD70 + ; percentage of CD138+ CD38+ myeloma cells were measured at the primary injection site (**K**), in the blood (**L**) and the contralateral tibial bone (**M**) 2 months post injection by flow cytometry; treatment was started at the day of injection (d0). Statistics: t-test (A, D, F–I, K–M), ELDA software https://bioinf.wehi.edu.au/software/elda/ [72] (B), one-way ANOVA with Tukey’s multiple comparisons test (E);*, *P* < 0.05; **, *P* < 0.01; ***, *P* < 0.001; ****, *P* < 0.0001; Data are shown as mean with SD.

Cell cycle analysis of freshly isolated EMM01 cells from a xenograft revealed higher proliferation rates in CD70+ cells, characterized by a lower fraction of cells in the G0/G1 phase and an increased percentage in the S/G2M phase compared to CD70- cells (Fig. 4A). When sorted CD70+ or CD70- EMM01 cells were injected subcutaneously in titrated numbers into NSG mice, the CD70+ fraction contained a significantly higher number of MM initiating cells (MMIC) (Fig. 4B).

To explore the direct impact of CD70/CD27 signaling, we generated CD70 knock-out (CD70KO) and AAVS1 control (AAVS1) KMS12-BM cell lines using CRISPR/Cas9 genome editing (Fig. 4C). CD70KO cells exhibited significantly higher CD27 expression, confirming that CD70/CD27 interaction is disrupted (Fig. 4D). Moreover, in vitro expansion of CD27KO and CD70/CD27 double KO (CD70KO/CD27KO) KMS12-BM cells was reduced to a similar extent as in CD70KO cells, indicating that the observed effects result from disruption of CD70/CD27 signaling (Fig. 4E). CD70 knock-out in a second cell line ARH77 also resulted in significantly reduced cell expansion compared to ARH77 AAVS1 control cells (Supplemental Fig. 4E). When treated with 41D12-D, AAVS1 control cells exhibited reduced expansion, while CD70KO and CD27KO cells showed no change (Supplemental Fig. 4F). Additionally, 41D12-D treatment increased the percentage of AAVS1 cells in the G0 phase and decreased the fraction of cells in the S/G2M phase, while there was no change in CD70KO and CD27KO cells (Supplemental Fig. 4G). Annexin-V staining showed no differences in apoptosis with 41D12-D treatment (Supplemental Fig. 4H).

To validate our findings in vivo, we subcutaneously injected KMS12-BM cells into NSG mice, followed by treatment with 41D12-D or IgG. 41D12-D significantly reduced tumor progression (Fig. 4F, G) and lowered sCD27 serum levels, indicating effective CD70/CD27 blockade (Fig. 4H). Moreover, primary EMM01 CD70+ cells injected s.c. into NSG mice failed to develop tumors when treated with 41D12-D, while IgG-treated controls formed tumors (Fig. 4I). CRISPR-Cas9-mediated CD70 knock-out in EMM01 cells similarly resulted in elevated CD27 surface expression and inhibited tumor engraftment and growth (Supplemental Fig. 4I–K). To determine whether the effect of CD70 blockade on MM growth is restricted to extramedullary disease or also applies within the bone marrow microenvironment, we injected primary EMM01 CD70+ cells intratibially and treated the mice with a CD70-blocking antibody. This treatment completely prevented intratibial tumor growth as well as dissemination into the blood and to the contralateral tibia (Fig. 4J–M).

Taken together, these findings highlight the importance of CD70/CD27 signaling in promoting MM cell expansion and progression in vivo.

### CD70/CD27 signaling induces MAPK/ERK and Wnt pathways in myeloma cells

**Fig. 5:**
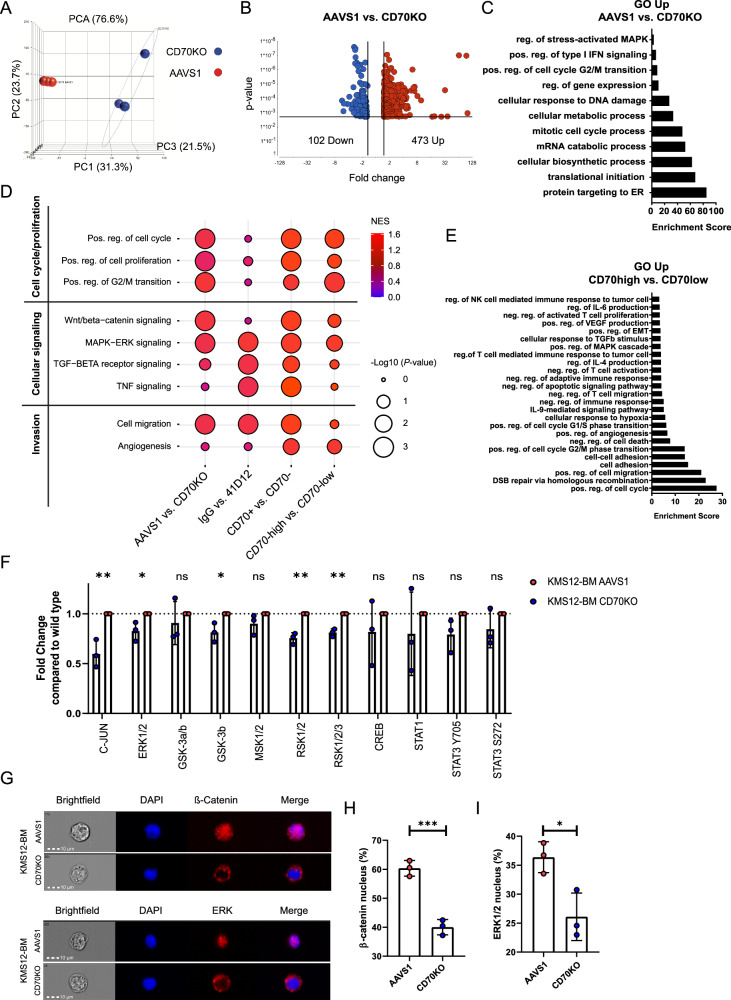
MAPK/ERK and Wnt signaling pathways are induced by CD70/CD27 signaling. **A** Principal component analysis (PCA) of EMM01 CD70KO versus CD70WT (AAVS1) tumors (*n* = 3 / group). **B** Volcano plot of differentially expressed genes in CD70WT (AAVS1) versus CD70KO myeloma cells. **C** Gene ontology (GO) analysis of upregulated genes (AAVS1 vs. CD70KO myeloma cells). GO enrichment score of ≥3 indicates significant changes. **D** Bubble plots illustrating significantly upregulated cell cycle and proliferation pathways, cellular signaling pathways or invasion related pathways within all four studied conditions based on gene set enrichment analysis and normalized enrichment scores (NES). **E** GO analysis of upregulated genes (*CD70* high vs. *CD70* low MM patients) from the CoMMpass study (identifier IA15). GO enrichment score of ≥3 indicates significant changes. **F** Phosphokinase array was performed in KMS12-BM AAVS1 and CD70KO cells; pooled data from *n* = 3 independent experiments are shown. **G** Nuclear localization of β-catenin and ERK1/2 was analyzed in KMS12-BM AAVS1 and CD70KO cell lines using co-localization with DAPI. **H**, **I** Percentage of nuclear β-catenin and ERK1/2 levels in KMS12-BM AAVS1 and CD70 KO cell lines; pooled data from *n* = 3 independent experiments are shown. Statistics: t-test (F, H-I);*, *P* < 0.05; **, *P* < 0.01; ***, *P* < 0.001; Data are shown as mean with SD.

To investigate the molecular mechanisms underlying CD70/CD27 signaling in MM, we performed RNA-seq on EMM01 CD70KO and AAVS1 control cells. Principal component analysis (PCA) demonstrated distinct clustering of CD70KO and AAVS1 cells, indicating clear transcriptional differences between the analyzed groups (Fig. 5A). Differential gene expression analysis identified 473 upregulated and 102 downregulated genes in AAVS1 compared to CD70KO cells (Fig. 5B). Gene Ontology (GO) analysis revealed that the upregulated genes were predominantly associated with cell metabolism, proliferation, and the regulation of gene expression (Fig. 5C). GSEA showed significant upregulation of MAPK/ERK and Wnt/β-catenin signaling pathways as well as cell proliferation, cell cycle transition, cell migration and angiogenesis in AAVS1 myeloma (CD70 WT) cells (Fig. 5D).

To corroborate these findings, we analyzed RNA-seq data from EMM01 myeloma cells treated in vivo with the blocking CD70 monoclonal antibody 41D12-D or IgG control. This analysis confirmed upregulated MAPK/ERK signaling as well as increased cell migration in IgG control vs. 41D12-D treated myeloma cells (Fig. 5D). GO analysis further showed upregulation of negative regulators of the MAPK/ERK signaling pathway and cytokine-related pathways, while genes involved in cell-cell and cell-matrix adhesion were significantly downregulated following CD70 blockade (Supplemental Fig. 5A, B).

Similarly, RNA-seq analysis of sorted CD70+ and CD70- fractions from EMM01 parental PDX samples revealed significant enrichment of genes associated with Wnt/β-catenin and MAPK/ERK signaling, cell proliferation, cell cycle transition, cell migration and angiogenesis in CD70+ myeloma cells (Fig. 5D). Supporting these observations, GSEA and GO results of -*CD70* high versus - *CD70* low MM samples from the CoMMpass study confirmed enrichment of genes linked to Wnt/β-catenin and MAPK/ERK signaling as well as cell proliferation, cell cycle transition, cell migration and angiogenesis (Fig. 5D, E). To validate our findings, we assessed the phosphorylation status of key protein kinases involved in major cellular signaling pathways in KMS12-BM CD70 KO and AAVS1 control cells using a phosphokinase array. In CD70 KO cells, we observed a significant reduction in the phosphorylation of ERK, GSK-3β, RSK1/2/3, and c-Jun, indicating diminished activation of the MAPK/ERK signaling cascade (Fig. 5F). Furthermore, CD70 KO cells showed markedly reduced nuclear translocation of ERK1/2 and β-catenin, suggesting impaired MAPK/ERK and Wnt/β-catenin downstream signaling upon disruption of CD70–CD27 interaction (Fig. 5G–I). In line with our previous findings, treatment of CD70-expressing MM cell lines with Wnt and MAPK inhibitors markedly reduced cell expansion and proliferation in KMS12-BM AAVS1 cells. In contrast, these effects were minimal or absent in CD70 KO cells (Supplemental Fig. 5C–E). These data indicate that CD70/CD27 engagement activates MAPK/ERK and canonical Wnt signaling in myeloma cells, thereby promoting enhanced cell-cycle progression and proliferation.

### Targeting CD70 with ADCC-optimized antibodies in MM

Our findings demonstrate that blocking CD70/CD27 signaling impairs myeloma progression. The selective expression of CD70 on activated lymphocytes and malignant cells provides a promising opportunity for targeted therapeutic interventions using cytotoxic agents. To explore this potential, we tested the efficacy of the antibody-dependent cell-mediated cytotoxicity (ADCC)-optimized antibody cusatuzumab, designed to target CD70-expressing myeloma cells in the presence of FcγRIIIA-expressing natural killer (NK) cells.

**Fig. 6:**
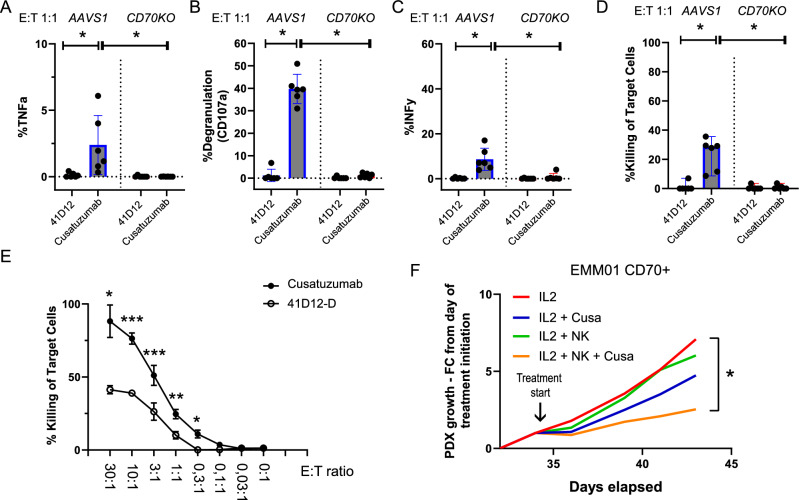
Treatment of MM with the ADCC-enhanced anti-CD70 antibody cusatuzumab. **A–D** Assessment of NK-cell activation upon co-culture of CD70WT (AAVS1) or CD70KO KMS12-BM cell lines in in the presence of 41D12 (Fc-dead) (10 μg/ml) or cusatuzumab with ADCC functions (10 μg/ml). **A** tumor necrosis factor alpha (TNFα), **B** CD107a, and **C** interferon gamma (IFNγ) were analyzed using flow cytometry; pooled data from *n* = 6 independent experiments. **D** Target cell specific killing of calcein-labelled CD70 WT (*AVVS1*) or *CD70* KO KMS12-BM cell lines analyzed upon co-culture with NK cells for 6 h in the presence of 41D12 or cusatuzumab (10 μg/ml); pooled data from *n* = 6 independent experiments. **E** Cusatuzumab-mediated myeloma cell lysis tested at different effector-to-target (E:T) ratios. **F** Treatment of EMM01 CD70+ tumor bearing mice with IL2 or IL2 plus cusatuzumab (Cusa) alone, NK cells alone or Cusa plus NK cells. 1.5×10^6^ PBMC-derived NK cells were injected intravenously when tumors reached a volume of 150mm3; simultaneously, intraperitoneal treatments with 41D12 or cusatuzumab (10 mg/kg body weight) were conducted every third day until the first tumor reached a size of 1000mm3; data from one experiment with *n* = 4 mice / group are shown. Statistics: Mann-Whitney test (A - D); t-test (E-F); *, *P* < 0.05; **, *P* < 0.01; ***, *P* < 0.001; Data are shown as mean with SD.

Treatment with cusatuzumab significantly enhanced NK-cell degranulation (as evidenced by CD107a expression) and increased the release of interferon-gamma (IFNγ) and TNFα compared to the CD70-blocking antibody 41D12-D. This effect was observed in the presence of CD70-expressing KMS12-BM, ARH77 and LP1 target cells but not in CD70KO controls (Fig. 6A–C and Supplemental Fig. 6A–C). Furthermore, cusatuzumab demonstrated superior efficacy in lysing KMS12-BM, ARH77 and LP1 CD70WT myeloma cell lines compared to 41D12-D, while no significant differences were observed in their CD70KO counterparts (Fig. 6D and Supplemental Fig. 6D). In addition, testing cusatuzumab at different effector-to-target (E:T) ratios revealed a ratio-dependent increase in myeloma cell lysis (Fig. 6E).

To assess the in vivo efficacy of cusatuzumab, EMM01 CD70+ tumor-bearing NSG mice were treated with cusatuzumab alone or in combination with peripheral blood mononuclear cell (PBMC)-derived NK cells, with interleukin-2 (IL2) supplementation. In the absence of NK cells, cusatuzumab exhibited a CD70-blocking effect that moderately reduced tumor growth compared to control groups. However, the combination of cusatuzumab with NK cells significantly suppressed tumor progression, demonstrating enhanced therapeutic efficacy (Fig. 6F).

In conclusion, our data indicate that CD70 can be effectively targeted using ADCC-enhanced antibodies such as cusatuzumab, offering a potent strategy to treat advanced myeloma.

## Discussion

MM is a hematologic malignancy characterized by an extensive intratumoral heterogeneity with the coexistence of multiple genetically and/or epigenetically distinct clonal subsets that expand differently during the disease course [2, 37]. During the course of MM progression, genomic instability in myeloma cells increases, partially driven by the tumor microenvironment or induced by anti-myeloma drugs [38–40]. In addition, autonomous signaling pathways or autocrine loops have been described that play a key role in the regulation of malignant plasma cell proliferation, therapy resistance and prevention of spontaneous and/or drug-induced cell apoptosis [3]. Here, we document that CD70/CD27 signaling is an autocrine loop that promotes the proliferation of myeloma cells.

CD70 overexpression has been reported in various B-cell malignancies such as mantle cell lymphoma, diffuse large B cell lymphoma or Waldenström macroglobulinemia where CD70/CD27 signaling is linked to rapid cancer progression, increased disease burden and dismal outcomes [17, 23, 41, 42]. CD70 overexpression on MM samples has been described before at various levels from 3 to 40% and has recently been linked to worse outcomes and distinct high-risk cytogenetic alterations by Kasap et al. [24, 25, 30]. In our large TMA containing over 200 MM biopsy specimens, we now documented that in 38. 3% of all MM specimens CD70 expression was detectable on clonal plasma cell populations but high expression profiles were found at a low frequency in early-stage disease. However, the percentage of CD70 expressing cells increases in late-stage disease with highest expression in EMM. Similarly, CD70 expression of primary MM transplanted as PDX in NSG mice increased after serial transplantations up to 100%. This suggests that CD70 expressing MM cells have a growth advantage and accumulate over time, independent of a MM-specific treatment. This however does not exclude that CD70 expression increases in response to treatment. Indeed, we previously documented that the CD70/CD27 interaction mediates resistance to treatment with tyrosine kinase inhibitors in CML [43]. Sorted CD70+ fractions required lower cell numbers to engraft in vivo in a limiting dilution assay, indicating a growth advantage and a higher disease initiating capacity. However, sorted CD70- cells re-expressed CD70 in vivo. The complete absence of CD70/CD27 signaling in transplantations of CD70KO cells prevented the growth of primary MM cells, indicating that this interaction is crucial for the propagation of primary MM cells in vivo.

CD70 expression on cancer cells has also been associated with higher cell proliferation [44–46]. Similarly, blocking CD70/CD27 signaling reduced the expression of genes associated with cell cycling and the proliferative capacity of MM cells. The induction of cell cycling and activation is mediated by CD27 receptor signaling leading to TNF Receptor Associated Factor 2/5 (TRAF2/5) activation and NF-ҡB pathway activation [17, 18]. In addition, we documented a link of TRAF2 to Wnt pathway activation via the downstream adaptor molecule TRAF2 and NCK Interacting Kinase (TNIK) [47]. CD27-signaling has been linked to induction of ERK1/2 and MAPK in primary plasma cell leukemia [29]. In accordance with these findings, Wnt and MAPK/ERK signaling was downregulated in sorted CD70- and in CD70KO myeloma cells. However, CD70 expression on CD27 negative cancer cells such as non-small cell lung cancer, mesothelioma and glioblastoma is also associated with worse prognosis due to epithelial-to-mesenchymal transition (EMT), enhanced migration and invasion [48–51]. The molecular mechanisms inducing EMT and migration in the absence of CD27 are unclear. In addition, expression of CD70 on cancer cells shapes the immune environment in the tumor due to the induction of CD27 signaling in tumor-infiltrating cells, i.e., CD70 expression induces the expansion of regulatory T cells in the tumor [52, 53]. In the present study, we focused on MM cell-autonomous effects of the CD70/CD27 interaction in vitro and in immune-incompetent mice in xenotransplantation experiments. This experimental setting does not allow to study effects of CD70 expression on MM on the modulation of the immune microenvironment.

We documented that CD70/CD27 signaling induced MAPK and Wnt signaling. Both pathways are crucial in myelomagenesis [54–56]. MAPK pathway activation in MM is often induced by mutations and genetic aberrations such as the t(4;14) translocation or mutations of *KRAS* or *NRAS* genes [56]. Interestingly, *RAS* mutations may drive the development of EMM [57]. In addition, external cues such as IL6, insulin-like growth factor (IGF) and, as shown in our study, CD70/CD27 signaling induce MAPK/ERK activation in MM [58–60]. Similarly, aberrant activation of Wnt induces the initiation and propagation of MM. BM stromal cells produce Wnt ligands that induce proliferation of MM cells [55]. We now show that the CD70/CD27 activation triggers Wnt signaling in the absence of the BM niche, probably driving extramedullary progression.

Although MM cases at early stages only contain a very low frequency of CD70 expressing cells, we found that CD70 expression is an independent negative prognostic marker in MM. CD70 expression in vivo was dynamically regulated. As previously reported, hypoxic environmental conditions and nutrient deprivation can alter the expression of distinct surface molecules such as CD138 on myeloma cells and thereby promote extramedullary dissemination [61–63]. In this context, hypoxia has also been documented to induce CD70 expression, which in turn has been linked to metastatic behavior in several solid cancer entities [32, 33, 51, 64, 65]. HIF1a is the main transcription factor in MM that induces CD70 expression. Other transcription factors such as SP1 in response to cell stress have been shown to lead to CD70 expression [43]. The expression of CD70/CD27 signaling on a subset of cells seems sufficient to induce MAPK/ERK signaling and Wnt/β-catenin pathway activation to promote myelomagenesis. Complete blockage of CD70/CD27 signaling by mAbs or CD70KO cells prevented myeloma growth.

MAPK and Wnt/β-catenin pathways have been identified as promising targets in the treatment of MM. Blocking the CD70/CD27 interaction reduced both these intracellular signaling pathways. However, the restricted selective and transient expression on activated lymphocytes, antigen-presenting cells and cancer cells makes CD70 a promising target for immunotherapy using ADCC-enhanced Abs recruiting NK cells, bi-specific Abs recruiting CD8^+^ T effector cells or even CAR-T cells. We found that the ADCC-enhanced anti-CD70 antibody cusatuzumab efficiently activates NK cells in the presence of CD70-expressing myeloma cells in vitro and that cusatuzumab treatment in combination with NK cells is efficacious in clinically established EMM in a xenotransplantation experiment. NK cell dysfunction is well-documented in relapsed/refractory MM, characterized by impaired cytotoxicity, reduced activation, and altered receptor expression [66–68]. Such deficits may limit the effectiveness of CD70-targeting immunotherapies, which rely in part on intact NK-cell–mediated antibody-dependent cytotoxic mechanisms. However, cusatzumab was designed with an ADCC-optimized Fcγ part and has been shown to overcome NK-cell dysfunction in AML [69]. How cusatuzumab might be included in combination therapies and how its efficacy compares to other immunotherapy approaches in MM requires further preclinical studies and ultimately a clinical trial. The data from the present study suggest that targeting CD70 might be most efficacious in late-stage, multi-resistant disease.

In summary, CD70/CD27 signaling activates MAPK/ERK and Wnt/β-catenin pathways in MM, both critical pathways for myelomagenesis especially in the development of EMM. Targeting CD70 using mAbs with enhanced effector function such as cusatuzumab is a potential therapeutic option particularly in the difficult to treat end stage MM.

## Supplementary information

Supplemental Data

## Data Availability

The *CD70* and *CD27* RNA-Seq data were extracted from the MMRF CoMMpass (versions IA15 and IA22) (https://research.themmrf.org and www.themmrf.org) In addition, *CD70* and *CD27* gene expression data were extracted from the cancer cell line encyclopedia (GSE36133). For original data, please contact the corresponding author adrian.ochsenbein@insel.ch.
