# Supplementary material for: CD70/CD27 signaling promotes the pathogenesis of multiple myeloma and represents a promising therapeutic target
Source: Leukemia. 2026 Mar 23;40(6):1163–75. doi: 10.1038/s41375-026-02899-1 (PMC13233305; doi:10.1038/s41375-026-02899-1)
Supplement: Supplementary file 1 — Supplemental Data [file 41375_2026_2899_MOESM1_ESM.docx]

**Supplemental Methods and Figures**

**CD70/CD27 Signaling Promotes the Pathogenesis of Multiple Myeloma and Represents a Promising Therapeutic Target**

Stefan Forster^1,2,3*^, Chantal Reinhardt^1*^, Maxime Boy^1*^, Adrian Wegmüller^1‡^, Alessio Hinrichsen^1‡^, Christian M. Schürch^4,5^, Frido K. Brühl^6^, Falko Fend^4^, Alexandar Tzankov^7^, Marie-Noëlle Kronig^2^, Michaela Römmele^1,2^, Angéline Glück^8^, Benjamin Lüscher^9^, Myriam Legros^9^, Ulrike Bacher^9^, Katja Seipel^2^, Thomas Pabst^2^, Ramin Radpour^1,2^, Carsten Riether^1,2^, Adrian F. Ochsenbein^2†^

*These authors contributed equally to this work as first authors

^‡^These authors contributed equally to this work as second authors

1 Tumor Immunology, Department for BioMedical Research (DBMR), University of Bern, Switzerland

2 Department of Medical Oncology, Inselspital, Bern University Hospital, University of Bern, Switzerland

3 Institute of Pathology, Technical University Munich, Munich, Germany

4 Department of Pathology and Neuropathology, University Hospital and Comprehensive Cancer Center Tübingen, Tübingen, Germany

5 Cluster of Excellence iFIT (EXC 2180) “Image-Guided and Functionally Instructed Tumor Therapies”, University of Tübingen, Germany

6 Department of Laboratory Medicine and Pathology, Ohiohealth, Columbus, Ohio, USA

7 Institute of Medical Genetics and Pathology, University Hospital Basel, Basel, Switzerland

8 Clinical Genomics Lab, Inselspital, Bern University Hospital, University of Bern, Switzerland

9 Department of Hematology and Central Hematology Laboratory, Inselspital, Bern University Hospital, University of Bern, Switzerland

†Correspondence: Department of Medical Oncology, Inselspital, Bern University Hospital and University of Bern, Switzerland; e-mail: [adrian.ochsenbein@insel.ch](mailto:adrian.ochsenbein@insel.ch)

**Supplemental Materials and Methods**

**Public Dataset Analysis**

Data from 766 patients from the Multiple Myeloma Research Foundation (MMRF) CoMMpass registry (NCT01454297), a prospective observational study where patients were followed every six months until death or censoring, were extracted. *CD70* TPM expression at baseline was analyzed in relation to overall survival (OS). Patients were stratified into *CD70*-high and *CD70*-low groups based on an optimal cut-off identified using the *survminer* R package function. Kaplan-Meier survival analysis, log-rank testing and visualizations were performed in R (v.4.2.0) using the packages survival, survminer, ggsurvfit, ggplot2 and ggbeeswarm.

**Cell culture**

The MM cell lines U266, RPMI 8226, SK-MM1, LP1, L363, MOLP2 and KMS12-BM as well as ARH77 cells were obtained from the German collection of microorganisms and cell cultures (DSMZ; Braunschweig). Cell lines were cultured according to the DSMZ guidelines in optimal culture media supplemented with 10% or 20% of fetal calf serum (FCS), 100 units/mL Penicillin/Streptomycin (Sigma-Aldrich) and 1% L-Glutamin (Sigma-Aldrich). All cell lines were kept in a 5% CO2 humidified atmosphere at 37°C. Cell lines were routinely tested for mycoplasma contamination and confirmed to be negative. Cell numbers were determined using trypan blue staining solution (Thermo Fisher) and the Countess II Automated Cell Counter (ThermoFisher). For hypoxia experiments, cells were incubated under normoxic culture conditions (21% O_2_; 5% CO_2_) or hypoxia (1.5% O_2_; 5% CO_2_) using a hypoxia workstation (Ruskinn). For blockade of HIF1a and HIF2a, the commercially available inhibitors PX-478 (MCE) and PT-2385 (MCE) were used, for inhibition of Wnt- and MAPK signaling, MM cells were treated with 10uM XAV939 (Sigma) and 10uM U0126 (Alomone labs), respectively.

**CRISPR-Cas9 Mediated *CD70* Gene Knockout**

Cas9:crRNA:tracrRNA ribonucleoprotein (RNP) was introduced via nucleofection into xenograft material or cell lines to generate a gene knock out. CRISPR RNAs (crRNAs) were designed using CHOPCHOP and CrispRGold. To correct for nuclease toxicity, a negative control crRNA targeting AAVS1, a safe harbor in the human genome, was designed^62,63^. Equimolar amounts of tracrRNA (IDT) and crRNA (IDT, *CD70*: CAGCTACGTATCCATCGTGA, AAVS1: GACGCAAGGGAGACATCCGT) were heated to 95°C and slowly cooled to room temperature. The crRNA:tracrRNA complexes were combined with Cas9 (TrueCut Cas9, ThermoFisher) in a 3:1 molar ratio. The mixture was incubated at room temperature for 10min to form RNP. Freshly isolated patient-derived xenograft cells were purified by FACS and incubated overnight in RPMI (Gibco) supplemented with 10% fetal bovine serum (FBS, Sigma), 100 units/mL Penicillin-Streptomycin (Sigma), and 1x GlutaMAX supplement (Gibco). Cells were washed with PBS and resuspended in 20uL buffer (P3 Primary Cell 4D-Nucleofector™ X Kit S, Lonza) at a concentration of 100’000 cells per reaction. 4uM Electroporation Enhancer (IDT) and 1ug of Cas9 in the form of RNP were added per reaction. Cells were incubated for 2min at room temperature before nucleofection in the 4D Nucleofector™ X Unit (Lonza) with pulse EO-100. Post-nucleofection, 200uL pre-warmed complete RPMI without antibiotics was added and cells were incubated for 20min at 37°C before transfer to a culture plate. After 6h, cells were counted and transplanted into NSG mice as described above. For *CD70* KO on cell lines, cells were split the day before the procedure. RNP was prepared as described above, using ATTO550-labelled tracrRNA (IDT). 1x10^6^ cells per reaction were washed with PBS and resuspended in 100uL buffer (Cell Line Nucleofector™ Kit V, Lonza). 5uM Electroporation Enhancer (IDT) and 5ug of Cas9 in the form of RNP were added per reaction. Cells were incubated for 2min at room temperature before nucleofection using the IIb Nucleofector™ (Lonza) with pulse X-001. Post-nucleofection, 500uL pre-warmed complete medium without antibiotics was added and cells were incubated for 20min at 37°C before transfer to a culture plate. After 24h, FACS-purification of the 20% ATTO550-highest live cells was performed. Cells were expanded and for cell lines completely positive for CD70, a pure CD70 KO population was achieved by FACS-purification of CD70-negative cells.

**HIF1α/HIF2α knockdown in SK-MM1 cells**

For short hairpin RNA (shRNA) induced knockdown experiments, 1x10^5^ SK-MM1 cells were seeded into a non-tissue culture treated 48-well plate and transduced with either an empty vector control (Santa Cruz Biotechnology) or with lentivirus expressing shRNA against *HIF1A* and *EPAS-1/HIF2A* alpha (Santa Cruz Biotechnology). Successfully transduced cells were selected by puromycin treatment.

**Analyses of gene expression using quantitative RT-PCR**

Quantitative real-time (qRT)-PCR was used to examine several candidate genes. Total RNA was extracted using the Quick-RNA MiniPrep kit (Zymo Research). High-Capacity cDNA Reverse Transcription Kit (Thermo Fisher Scientific, Switzerland) was used to create cDNA. Primerquest Software (Integrated DNA Technologies) or Primer3Plus (http://www.bioinformatics.nl/) were used to design primers for each candidate gene. FastStart Universal SYBR® Green 2X PCR Master Mix (Roche, Switzerland) was used for qRT-PCR. The qRT-PCR reactions were carried out in replicates using an Applied Biosystems QuantStudio 3 System.

**Immunohistochemistry stainings**

Formalin-fixed and paraffin-embedded (FFPE) tissues and unstained tissue microarray sections were obtained from MM patients with informed consent and were provided by the Institutes of Pathology in Basel and Bern (Switzerland) and Tübingen (Germany). From FFPE tissues, sections were cut at 2μm thickness. Stainings for MUM1 (clone MUM1p; Agilent Dako; dilution 1:200; incubation in TrisEDTA for 40 minutes at 95°C), CD70 (clone 301731; R&D systems; dilution 1:200; incubation in citrate for 30 minutes at 100°C), CD27 (clone LPFS2/1611; Abcam; dilution 1:500; incubation in citrate for 20 minutes at 95°C) and HIF1α (clone GT10211; GeneTex; dilution 1:300; incubation in citrate for 20 minutes at 95°C) were conducted using a Leica BOND RX automated immunostainer (Leica Biosystems) followed by visualization with DAB.. CD70 and CD27 expression were analyzed on MUM1 positive infiltrating plasma cell populations. For quality control and scoring of CD70 and CD27 expression levels, tissue slides were analyzed by one pathologist in training (SF) supervised by two board-certified pathologists with specialization in hematopathology (FKB and CMS). Scoring was performed for both CD70 and CD27 accordingly: Score 0 (negative; absence of expression), 1 (low; less than 3% positive plasma cells), 2 (moderate; ≥ 3% - 10% positive plasma cells) and 3 (high; ≥ 10% positive plasma cells). All slides were scanned and digitally evaluated using the Pannoramic P250 Flash III digital slide scanner and SlideViewer software (3DHistech).

**Fluorescence activated cell sorting, antibodies and compounds**

*FACS antibodies* used: αCD70-PE (Clone Ki-24; BD Bioscience), αCD27-FITC (Clone LG.7F9; Biolegend), αCD138-BV605 (Clone MI15; BD Bioscience), αCD38-APC (Clone HIT2; Biolegend), PE mouse IgG3, ҡ isotype control (Clone A112-3; BD Bioscience), FITC Armenian Hamster IgG isotype control (clone HTK888; Biolegend); eBioscience fixable viability dye eFluor 450 (Thermo Fisher); Annexin V-PE (Biolegend). Flow cytometry analyses were performed on a BD Fortessa and cells were sorted with a MoFlo ASTRIOS BSL-2 cell sorter (Beckman Coulter). All data were collected and analyzed using FACSDiva software (BD Pharmingen) and FlowJo software (Tree Star Inc.), respectively. Human anti-CD70 monoclonal antibody 41D12-D and ADCC-enhanced human anti-CD70 antibody cusatuzumab were provided by ArgenX. Palivizumab ([Synagis]; AstraZeneca) was used as control treatment for 41D12-D. For *in* *vitro* studies cells were treated with 10μg/ml of CD70 targeting antibodies or respective IgG controls. For *in vivo* treatments 10mg/kg of 41D12-D, cusatuzumab or IgG control antibodies were used.

**Immunofluorescence.**

KMS12-BM AAVS1 and CD70KO cells were fixed in 4% paraformaldehyde, blocked with 10% goat serum, and stained with primary antibodies against active β-catenin (rabbit anti-β-catenin, D13A1, dilution 1:800) or D13.14.4E rabbit anti-ERK1/2 (dilution 1: 200) overnight at 4°C. Secondary staining was performed using goat anti-rabbit AF647 (dilution 1:1000), followed by DAPI nuclear counterstaining. Compensation and fully stained samples were prepared in biological triplicates. Samples were acquired using the ImageStream®X Mark II Imaging Flow Cytometer. Subsequent analysis was performed with the IDEAS® image analysis software using the Nuclear Localization Wizard to quantify the percentage of cells displaying nuclear ERK or β-catenin localization through co-localization with DAPI. Data acquisition was performed on an Image Stream system and nuclear localization was analyzed using co-localization with DAPI.

**Detection of soluble sCD27 in sera**

Soluble CD27 levels in sera from xeno-transplanted NSG mice were determined using CD27 (Soluble) Human Instant ELISA™ Kit (Thermo Fisher). Assays were performed according to the manufacturer’s instructions.

**Phosphorylation array**

KMS12-BM AAVS1 and CD70KO cell lines were processed using the Human Phospho-Kinase Array Kit (R&D systems, ARY003C) according to the manufacturer’s instructions.

**High-throughput transcriptome analysis using next generation RNA sequencing (RNA-Seq)**

Total RNA was extracted from 41D12-D or IgG treated and FACS-purified, CD70 KO and AAVS1 (CD70 wild type) as well as CD70+ and CD70- myeloma cells using the RNeasy Micro Kit (QIAGEN AG) according to the manufacturer's instructions. The Bioanalyzer instrument (Agilent Technologies) and the RNA Pico Chip (Agilent Technologies) were used to perform a quality control check on the extracted RNA. A Quantus Fluorometer (Promega) and the QuantiFluor RNA system kit (Promega) were used to quantify RNA. The SMART-Seq Ultra Low Input RNA Kit for Sequencing (Takara Bio, USA) was used to prepare libraries from total RNA. The libraries were tested for quality using the Fragment Analyzer and the High Sensitivity NGS Fragment Analysis Kit (Agilent, Germany). Libraries were sequenced using the NextSeq 500 High Output Kit 75-cycles (Illumina, USA) loaded at 2.0pM and 1% PhiX. The Illumina RTA version 2.11.3 and Basecalling Version bcl2fastq-2.20.0.422 were used for primary data analysis. The RNA-Seq data was assembled with the use of GRCh38 human genome reference template. TMM (edgeR) normalization and log2 transformation were used to determine the level of gene expression. Two-way ANOVA was used to evaluate the data set. Following statistical analysis, genes having a significant difference in expression at FDR < 0.05 and fold differences of at least 1.5 were chosen. The variances among profiled samples were mapped using principal component analysis (PCA). The data was grouped using the conventional Euclidean approach based on average linkage, and heatmaps were created using the typical normal distribution of the values.

**Gene ontology and gene set enrichment analysis**

The differentially expressed genes were organized into functional hierarchies for gene ontology (GO) enrichment. Using KEGG Mapping (www.genome.jp/kegg) and the Enrichr database (https://amp.pharm.mssm.edu/Enrichr), enrichment scores were obtained by comparing the proportion of the gene list in a group to the proportion of the background genes. A score of 3 or higher indicated a significant enrichment (*P*<0.05). Gene set enrichment analysis (GSEA) was carried out using GSEA software v.4 (Broadinstitute, Cambridge). All pathway-related genes were analyzed for enrichment using the Pathcards database (pathcards.genecards.org), GO datasets (geneontology.org), and the Broad Institute's Molecular Signatures Database (MSigDB).

**NK cell isolation and co-culture experiments**

NK cells were isolated from peripheral blood mononuclear cells (PBMC) using an NK cell isolation kit (Miltenyi Biotec 130-092-657). NK cell purity was assessed by flow cytometry using anti-CD3, anti-CD56 and anti-CD16 antibodies. Populations with at least 95% of CD56^+^CD3^-^ isolated cells were considered pure NK cells. To assess NK cell activation in the presence of CD70 expressing target cells, NK cells were incubated overnight at 37°C in the presence of 100UI/mL IL-2. Afterwards, NK cells were co-cultured with CD70KO or CD70 wild type MM cell lines at different effector-to-target (E:T) ratios in presence of the antibody constructs cusatuzumab or 41D12-D (at 10 µg/ml) and anti-CD107a-PE-Cy7 antibodies (BD 561343) for 6 hours. After 1 hour of incubation brefeldin A (Sigma) was added at a final concentration of 10 µg/ml. The percentages of CD107a and IFN-γ positive cells were estimated by flow cytometry analysis within CD56^+^ CD3^−^ NK cell populations. Spontaneous degranulation and cytokine release in the absence of target cells were used as control settings. NK cell mediated killing of target cells was analyzed by monitoring calcein release. Briefly, NK cells were incubated overnight at 37°C in the presence of 100UI/ml IL-2 (PeproTech). Next, NK cells were collected, enumerated, and co-cultured with MM cell lines that had been calcein-labeled in 96-well U-bottom plates in media supplemented with Probenecid at a 1:1 ratio in the presence of cusatuzumab or 41D12-D at 10 µg/ml. Target cell killing was quantified after 4 hours of incubation measuring calcein-release in supernatants. Target cell specific killing was calculated as: Measured fluorescence of target cells + spontaneous fluorescence - spontaneous fluorescence) / (maximum fluorescence - spontaneous fluorescence)*100. Target cells treated with NP-40 served as positive control for maximum fluorescence levels and target cells treated with antibodies but without effector cells served as negative controls for spontaneous fluorescence. For *in vivo* studies, 1.5x10^6^ PBMC-derived NK cells were intravenously injected in tumor bearing NSG mice when tumors reached an average volume of 150mm^3^. Subsequently, mice were intraperitoneally treated with cusatuzumab (10mg/kg) and/or Interleukin 2 (50’000 UI) every third day until the first tumor reached a size of 1000mm^3^.

**Statistical analysis**

All data were analyzed using GraphPad software (GraphPad Prism v7). Extreme limiting dilution analysis (ELDA) was used to calculate stem cell frequency and tumor initiation capacity (software: https://bioinf.wehi.edu.au/software/elda/)^64^. Data are displayed as mean with standard deviation (SD). Statistical tests were used as indicated in the figure legends. *P*-values < 0.05 were considered significant.

**Supplemental Figures**


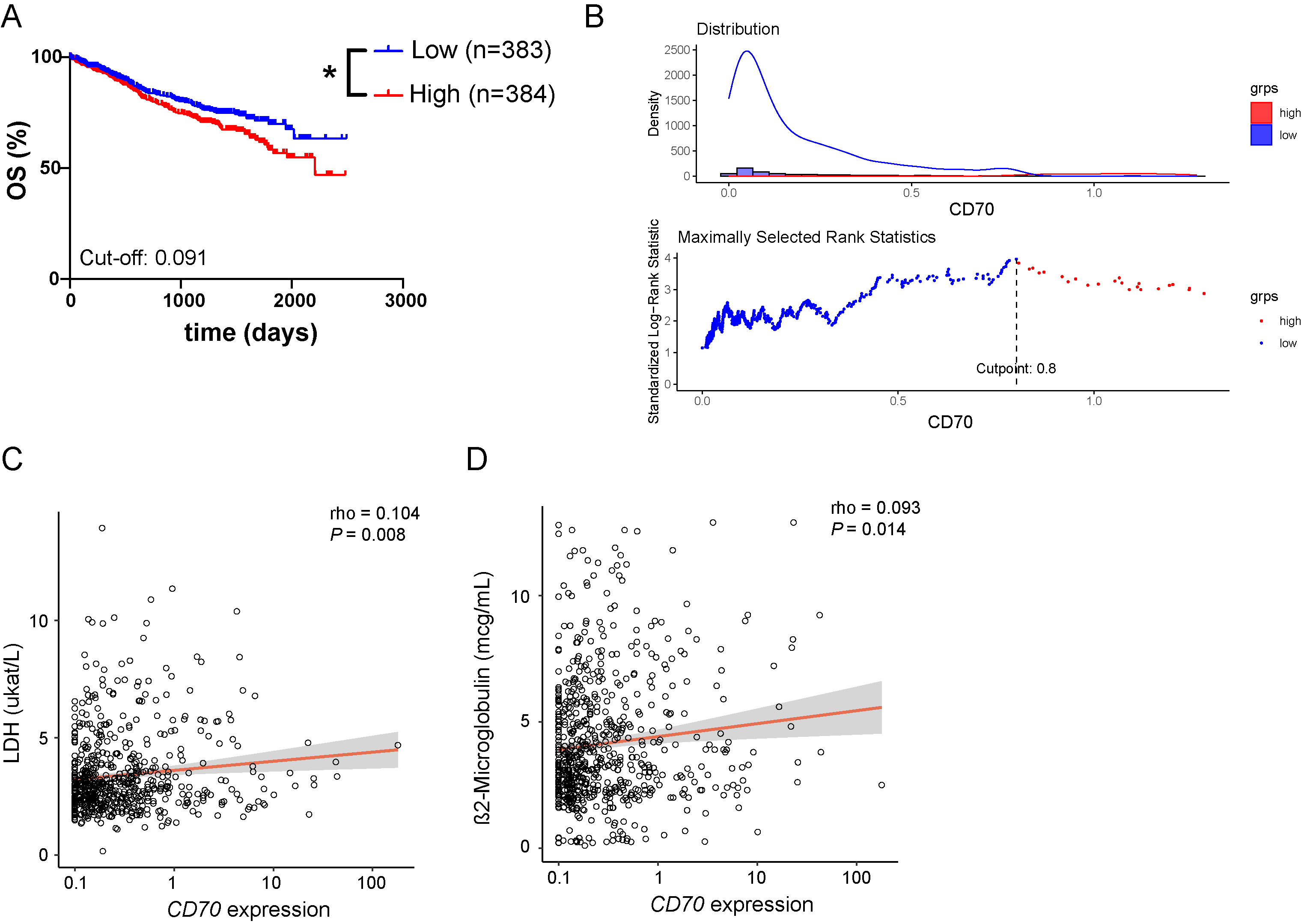


**Supplemental Fig. 1:** **A** Kaplan-Meier survival curve of *CD70* high (n = 384) versus *CD70* low (n = 383) (separated by the median-cut off) MM patients (data were extracted from the CoMMpass database; identifier IA15). **B** Maximally selected rank statistics were applied for optimal cut-off determination (data were extracted from the CoMMpass database; identifier IA15). **C-D** Correlation analyses between LDH (C) and β2 – Microglobulin (D) serum levels and *CD70* expression (data were extracted from the CoMMpass study; identifier IA15). Statistics: log-rank test (A), Spearman’s rho test (C-D); *, *P* < 0.05; Data are shown as mean with SD. **Abbreviations:** LDH, lactate dehydrogenase; MM, multiple myeloma; SD, standard deviation; β2-microglobulin, beta-2 microglobulin.


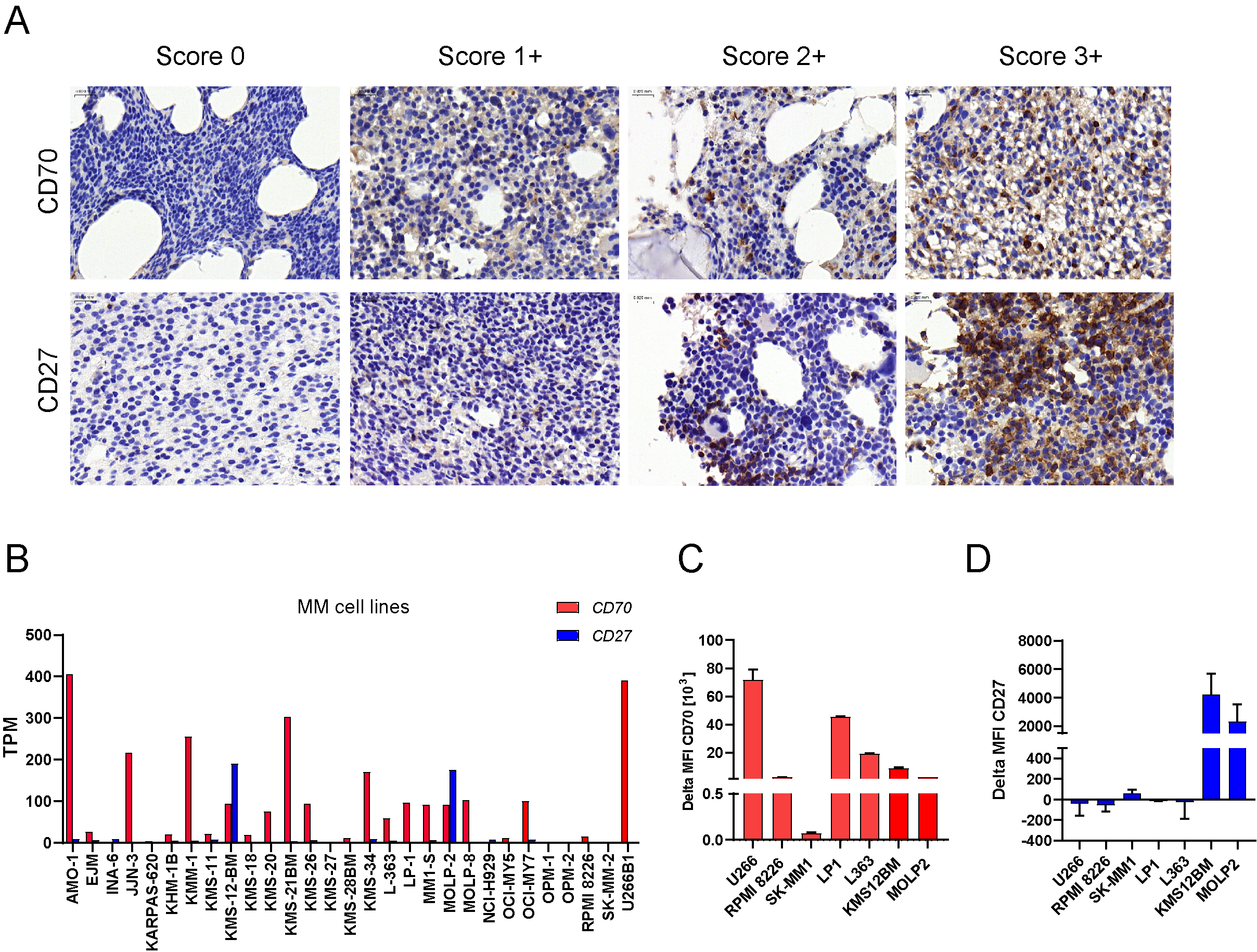


**Supplemental Fig. 2:** **A** Scoring system (Score 0 – 3+) based on the percentages of CD70 or CD27 expressing myeloma cells. Representative images of each score are shown. **B** RNA sequencing data of 29 MM cell lines from the CCLE; data were analyzed for *CD70* and *CD27* mRNA expression levels (TPM). **C-D** Delta MFI of CD70 (C) and CD27(D) versus the respective isotype controls in seven different MM cell lines (U266, RPMI8226, SK-MM1, LP1, L363, KMS12-BM, and MOLP2); pooled data from n = 2 independent experiments are shown. Data are shown as mean with SD. **Abbreviations:** CCLE, Cancer Cell Line Encyclopedia; MFI, median fluorescence intensity; MM, multiple myeloma; mRNA, messenger RNA; SD, standard deviation; TPM, transcripts per million.


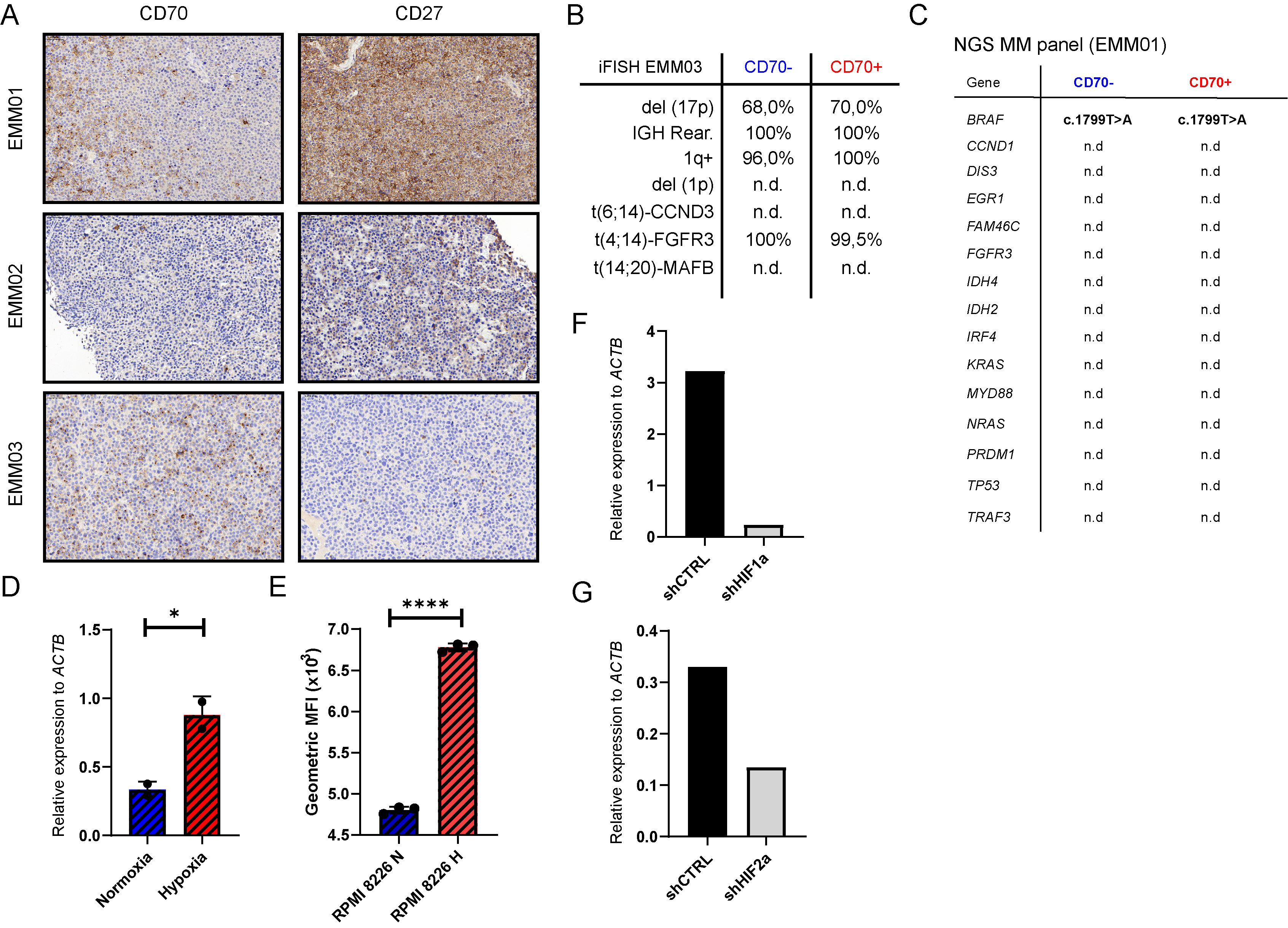


**Supplemental Fig. 3:** **A** CD70 and CD27 immunohistochemistry stainings of the primary MM specimens EMM01, EMM02 and EMM03 showing CD70 and CD27 (co-) expression. **B** iFISH analysis for distinct cytogenetic alterations in EMM03-derived CD70+ and CD70- myeloma cell fractions. **C** Comparative analysis between EMM01 derived CD70+ and CD70- myeloma cell fractions for hotspot mutations in oncogenic driver genes via next generation sequencing. **D** *CD70* mRNA expression levels were analyzed in SK-MM1 cells under normoxic or hypoxic culture conditions over 120 hours using qRT-PCR; data are displayed as ΔCt normalized to *ACTB* housekeeping gene expression. **E** CD70 upregulation under hypoxic culture conditions in RPMI 8226 myeloma cells. **F-G** *HIF1A and EPAS-1/HIF2A* knockdown status in SK-MM1 cells was confirmed using qRT-PCR; data are displayed as ΔCt normalized to *ACTB* housekeeping gene expression in comparison to shRNA control treated SK-MM1 cells. Statistics: t-test (D, E); *, *P* < 0.05; ****, *P* < 0.0001; Data are displayed as mean with SD. **Abbreviations:** ACTB, beta-actin; EMM, extramedullary myeloma; EPAS1, endothelial PAS domain protein 1; HIF, hypoxia-inducible factor; iFISH, interphase fluorescence in situ hybridization; MM, multiple myeloma; mRNA, messenger RNA; n.d., not detected; qRT-PCR, quantitative real-time polymerase chain reaction; SD, standard deviation; shRNA, short hairpin RNA.

**
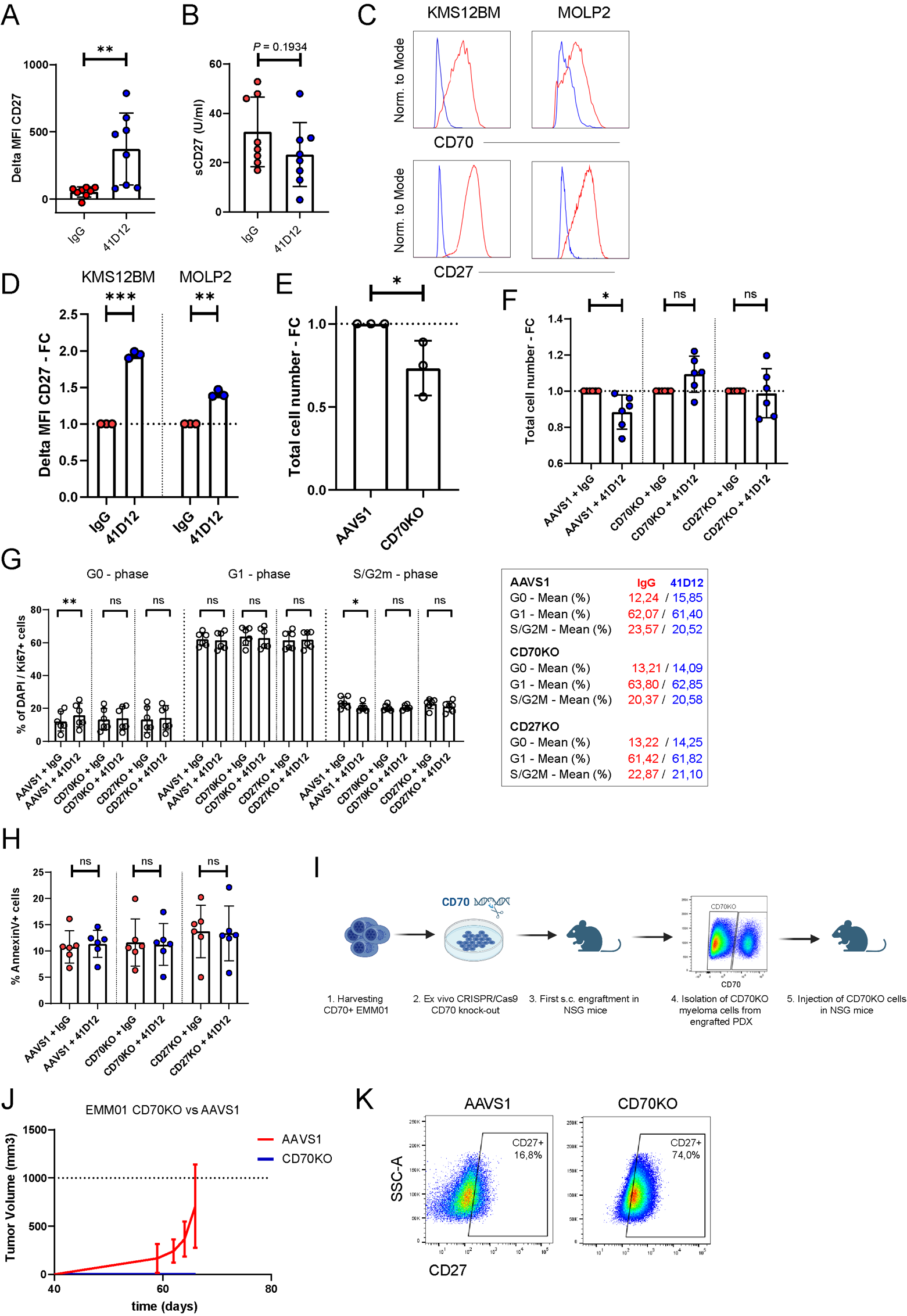
Supplemental Fig. 4:** **A** Delta MFI of CD27 expression versus isotype control upon treatment with 41D12-D (41D12) or IgG control antibodies in EMM01 CD70+ xenografts; pooled data from two independent experiments with n = 8 mice per group. **B** Soluble CD27 levels were measured in sera of mice bearing CD70+ EMM01 xenografts upon treatment with 41D12 or IgG (n = 8 mice / group). **C** CD70 and CD27 protein expression levels on KMS12-BM and MOLP2 cell lines analyzed by flow cytometry. Blue curve: isotype staining, red curve: CD70 or CD27 staining. **D** FC of Delta MFI of CD27 protein expression versus isotype control upon treatment with 41D12-D (41D12) or IgG control antibodies in KMS12-BM and MOLP2 MM cell lines; pooled data from n = 3 independent experiments. **E** Total cell numbers of ARH77 AAVS1 versus ARH77 CD70KO cells, pooled data from n = 3 independent experiments. **F** Total cell numbers of KMS12-BM AAVS1, CD70KO or CD27KO upon treatments with 41D12-D (41D12) or IgG control antibodies were determined after 72 hours of incubation period using FACS counting beads; data shown as fold change to IgG control treatments; pooled data from n = 6 independent experiments. **G** DAPI/Ki67 staining 72h after 41D12 or IgG treatment of KMS12-BM AAVS1, CD70KO or CD27KO cells; pooled data from n = 6 independent experiments are shown. **H** Percentages (%) of AnnexinV+ apoptotic cells were determined by flow cytometry in KMS12-BM AAVS1, CD70KO or CD27KO cells upon treatment with 41D12-D (41D12); pooled data from n = 6 independent experiments. **I** Graphical scheme illustrating the generation of EMM01 derived CD70KO myeloma cells (image created in BioRender). **J** Engraftment of CD70KO EMM01 myeloma cells compared to AAVS1 control cells; n = 2 mice injected with CD70KO cells and n = 3 mice injected with AAVS1 control cells. **K** CD27 surface expression in EMM01 AAVS1 or CD70KO myeloma cells measured by flow cytometry. Statistics: t-test (A, B, D and E), One-way ANOVA with Tukey’s multiple comparisons test (F-H); *, *P* < 0.05; **, *P* < 0.01; Data are shown as mean with SD. **Abbreviations:** DAPI, 4′,6-diamidino-2-phenylindole; EMM, extramedullary myeloma; FACS, fluorescence-activated cell sorting; FC, fold change; IgG, immunoglobulin G; KO, knock-out; MFI, median fluorescence intensity; MM, multiple myeloma; SD, standard deviation.


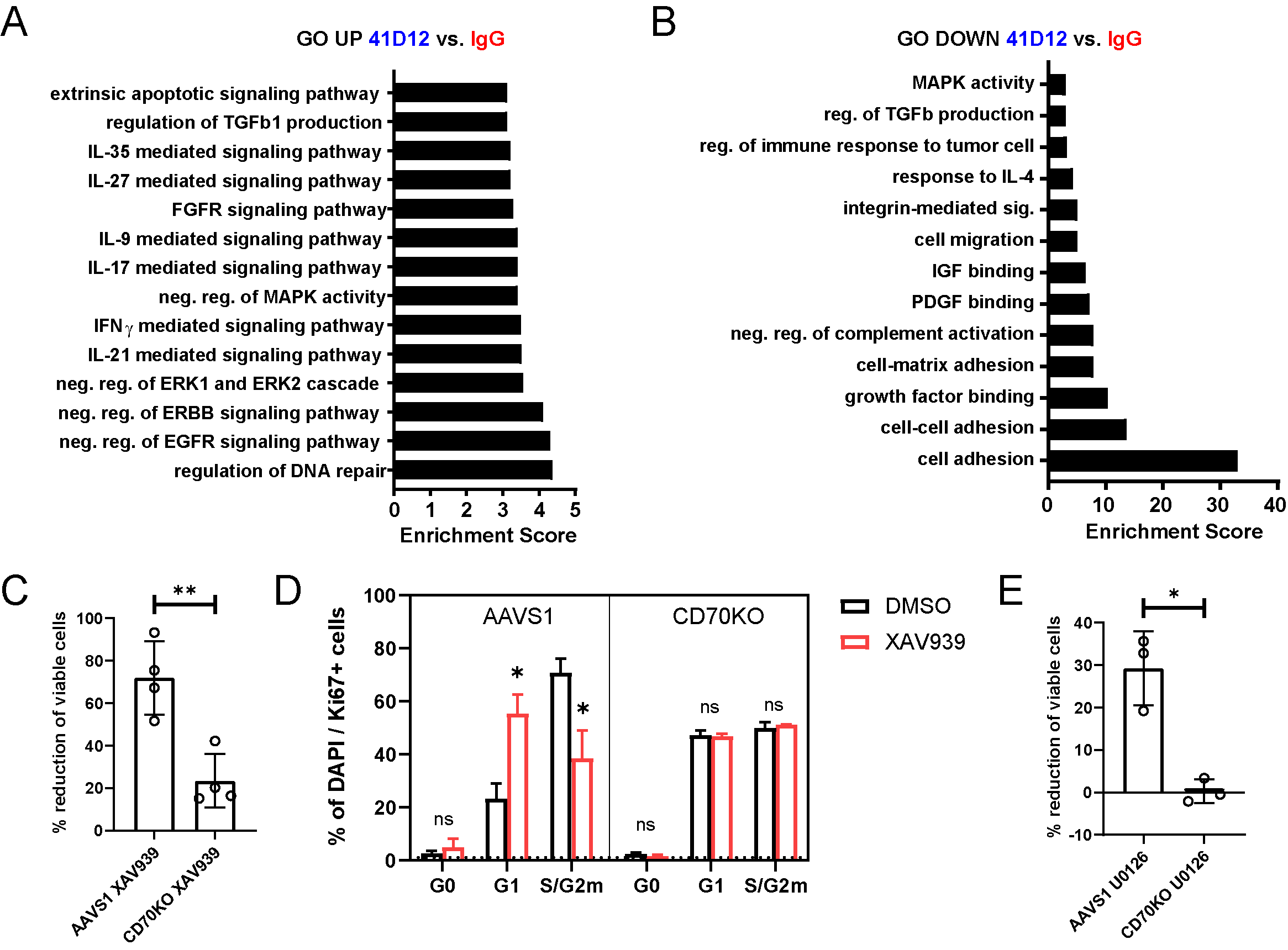


**Supplemental Fig. 5:** **A-B** GO analysis of upregulated (A) and downregulated (B) pathways and biological processes in 41D12 vs. IgG treated myeloma cells. GO enrichment score of ≥3 indicates significant changes. **C** Cell expansion of AAVS1 or CD70KO KMS12-BM cells upon treatment with the Wnt-inhibitor XAV939. **D** Cell cycle states of AAVS1 or CD70KO KMS12-BM cells upon treatment with the Wnt-inhibitor XAV939. **E** Cell expansion of AAVS1 or CD70KO KMS12-BM cells upon treatment with the MAPK-inhibitor U0126. Statistics: t-test (C, E), One-way ANOVA with Tukey’s multiple comparisons test (D); *, *P* < 0.05; **, *P* < 0.01; Data are shown as mean with SD. **Abbreviations:**  GO, gene ontology; KO, knock-out; MAPK, mitogen-activated protein kinase; SD, standard deviation.


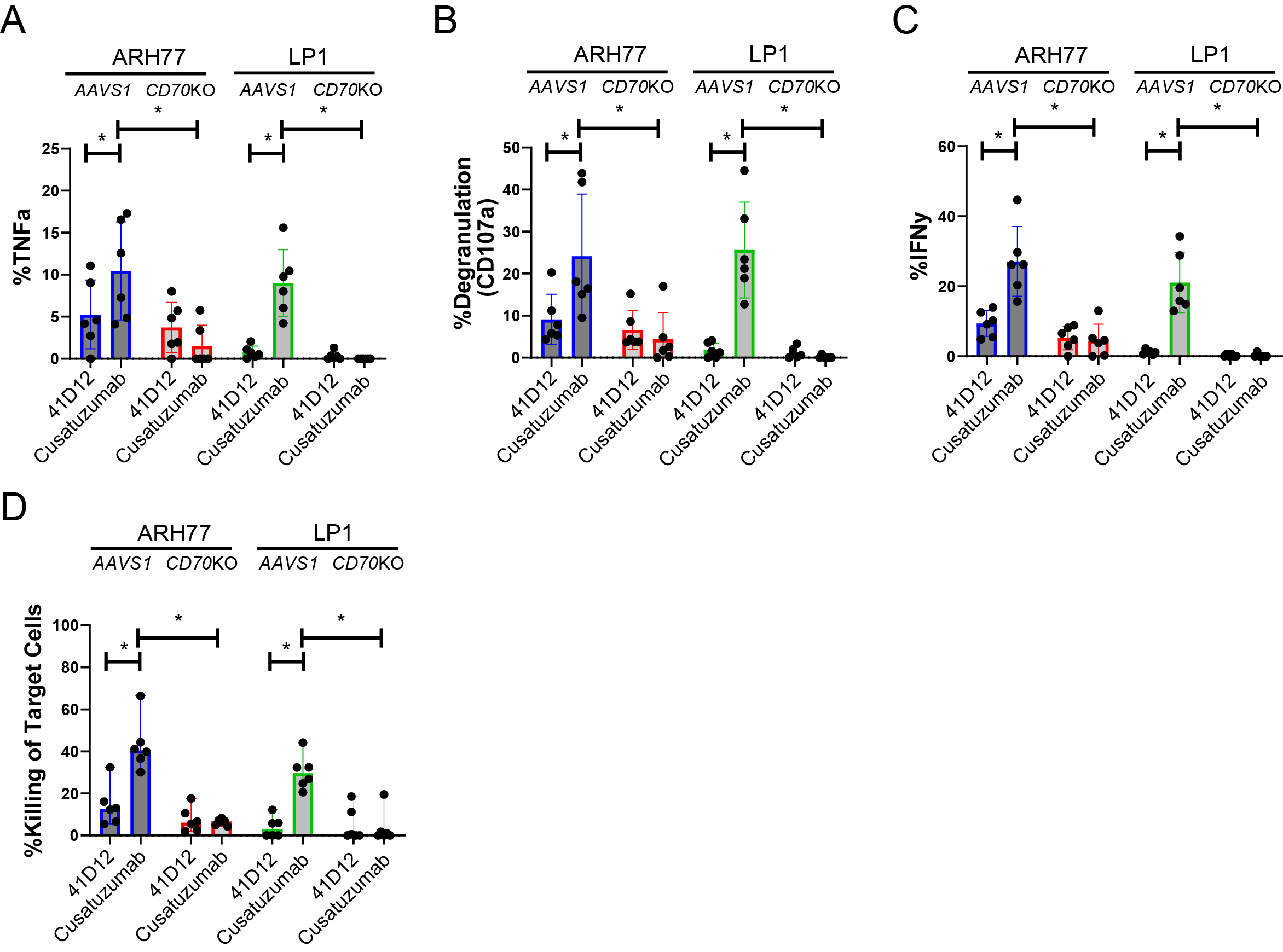


**Supplemental Fig. 6:** **A-C** Assessment of NK-cell activation upon co-culture of AAVS1 or CD70KO cell lines (ARH77 and LP1) in in the presence of 41D12 (Fc-dead) (10μg/ml) or cusatuzumab with ADCC functions (10μg/ml). (A) TNFα, (B) CD107a, and (C) IFNγ were analyzed using flow cytometry; pooled data from n = 6 independent experiments. **D** Target cell specific killing of calcein-labelled AAVS1 or CD70 KO cell lines (ARH77 and LP1) analyzed upon co-culture with NK cells for 6 hours in the presence of 41D12 or cusatuzumab (10μg/ml); pooled data from n = 6 independent experiments. Statistics: Mann-Whitney test (A - D); *, *P* < 0.05; Data are shown as mean with SD. **Abbreviations:** ADCC, antibody-dependent cellular cytotoxicity; IFNγ, interferon gamma; KO, knock-out; NK, natural killer; SD, standard deviation; TNFα, tumor necrosis factor alpha.
